# Small bowel metastases from a testicular choriocarcinoma revealed by gastrointestinal bleeding: a case report

**DOI:** 10.11604/pamj.2021.39.259.30325

**Published:** 2021-08-20

**Authors:** Cherihane Dassouli, Chama El Manjra, Adil Ait Errami, Sofia Oubaha, Zouhour Samlani, Khadija Krati

**Affiliations:** 1 Department of Gastroenterology, Mohamed VI University Hospital, Marrakech, Morocco

**Keywords:** Testicular choriocarcinoma, small bowel metastases, melenas, intussusceptions, case report

## Abstract

Testicular germ cell tumors are the most common cancers in young men. In most cases, patients may present a painless testicular swelling. However, in 10% the presentation is variable and related to site of metastasis and complications. Clinically apparent gastrointestinal involvement was seen in 5% of cases and dominates by gastrointestinal bleeding. We report a case of testicular choriocarcinoma involving the small intestine revealed by melena and complicated by acute intussusception.

## Introduction

Testicular cancers are the most common malignant neoplasm in men aged 15-35, but only constitute 1% of cancers in males [1]. Germ cell tumors (GCTs) account for more than 90% of testicular cancer. Clinically, these tumors are classified as either seminoma or non-seminoma [2]. In most cases, GCTs present as a painless testicular swelling. However, in 10% of patients, presentation is variable and related to site of metastasis and complications [3]. These tumors typically metastasize to the retroperitoneal lymph nodes and less frequently to the lungs, liver and brain [4], while Gastrointestinal (GI) metastasis is seen in 5% of germ cell tumors and rare presentation with gastrointestinal bleed have been reported, with small intestine involvement seen in only 1.4% of cases [2]. We report a case of testicular choriocarcinoma involving the small intestine revealed by melena and complicated by acute intussusceptions.

## Patient and observation

**Patient information:** a 33-year-old, with no medical history besides 5-pack-year history of tobacco use, has been admitted to our emergency department with melena of 3 weeks duration. He denied hematemesis, abdominal pain, changes in bowel habits or vomiting.

**Clinical findings:** on physical exam, the patient was normotensive, afebrile and has normal heart rate. He had significant pallor. The abdominal exam was benign and the rectal exam showed melena. Left testicular swelling was noted, which had become hard on palpation with negative transillumination test.

**Diagnostic assessment:** laboratory studies showed anemia with Hb of 8.7g/dL. Esophagogastroduodenoscopy was done urgently and showed a gastritis. No active bleeding was seen. A scrotal ultrasound was performed, revealing a left testis measuring 6x5.2x6 cm with a heterogeneous echo texture and multiple nodular lesions suspicious for neoplasm. The patient's β-human chorionic gonadotrophin level was elevated at 805588IU/mL. Lactate dehydrogenase level was also elevated at 699 U/L, and serum α-fetoprotein level was normal at 2.8 ng/ml. The following day, the patient developed serious abdominal pain with bilious vomiting. An urgent computerized tomography was done and showed a small bowel intussusception due to an umbilicated lesion measuring 4x3cm, with multiple liver, lung and left kidney nodules.

**Therapeutic intervention:** the patient underwent left orchiectomy with intestinal resection, removing the umbilicated lesion and biopsy of the liver nodule. Histologic studies of the left testis, intestinal and hepatic specimens removed revealed choriocarcinoma.

**Follow-up and outcomes:** the multidisciplinary team decision was an aggressive chemotherapy, but unfortunately, the patient died few days after.

## Discussion

Testicular cancers, though rare (1%), represent the most common neoplasm among young men. It is more common in white males than black males [1]. Dominated by par germ cell tumors that can be seminomas or non-seminomas. Testicular choriocarcinomas are highly aggressive due to their rapid proliferation, excessive vascularity and neovascularization leading to usual tumor necrosis [4]. Most testicular tumors present as a painless mass, but can be painful if bleeding or infarction occurs. However, they can be discovered through atypical symptoms related to metastasis and organs involved [5].

Gastrointestinal metastases were found in 27% of testicular cancer cases in a postmortem study [3]. In a necropsy series of 990 patients with testicular tumors, Dixon and Moore identified 7 cases of pure choriocarcinoma, 5 of which were associated with metastases to the gastrointestinal tract. However, few of these metastases were clinically apparent [6]. The gastrointestinal involvement is commonly associated with non- seminomatoustumors [7]. Metastases to the gastrointestinal tract occur by either direct tumor extension from affected lymph nodes or hematogenous spread. The sites of GI involvement include the duodenum, jejunum, ileum, stomach, esophagus, colon and the pancreas. The duodenum is the most common site of involvement because of its anatomic proximity to the retroperitoneal nodes, where the lymphatic drainage of the testis occurs [8].

But clinically apparent gastrointestinal involvement was seen in < 5% of cases, and presents as abdominal pain (46%), melena (44%), hematemesis (24%), vomiting or abdominal mass [3]. Clinical digestive symptoms are mostly related to intestinal obstruction, pancreatic involvement, or mucosal ulcerations. The symptoms of anemia secondary to GI blood loss can appear. The blood loss can be occult or massive. Johnson *et al*. [6] found a hemorrhagic complication in 7 of 16 patients with choriocarcinoma. Of these 7 patients, 4 died of massive GI hemorrhage. Choriocarcinoma generally has rapid proliferation, excessive vascularit and a tendency to outgrow its blood supply. Choriocarcinomas with these characteristics, therefore, have a tendency to ulceration, necrosis and then bleeding. Necrosis resulting from chemotherapy is another frequent cause of GI bleeding, which can be fatal [9].

In patients with testicular cancer, especially those with disseminated choriocarcinoma, gastrointestinal involvementshould be considered if symptoms of gastrointestinal bleeding develop [10]. The patients with gastrointestinal presentation of their germ cell malignancy most often belong to a poor-prognosis group, due to large-volume disease elsewhere, independent of histology. They are today treated with extremely intensive chemotherapy [4]. Platinum-based chemotherapeutic regimens have high success rates in the treatment of metastatic germ cell tumors [11]. But the outcome of the patients with gastrointestinal involvement is quite poor and source of failure after chemotherapy. Therefore, early intervention with surgical resection of the involved segment of bowel is necessary when gastrointestinal complications develop [7].

Although there have been reports in the literature of germ cell tumors metastasizing to the gastrointestinal tract, they have generally been in the setting of known primary testicular cancer or metastatic disease. The specification of our case is the diagnostic of testicular choriocarcinoma, which wach until then ignored, a small intestine metastasis revealed by melena and complicated by acute intussusception.

## Conclusion

Testicular germ cell tumors are a malignancy of the young. Young men presenting with evidence of gastrointestinal bleed with suggestions of underlying malignancy may not offer information on testicular swelling, even if present. A review of systems that includes scrotal/testicular swelling should be conducted and a genital examination done if appropriate. An early recognition of germ cell malignancy localized in the upper GI tract and adequate multimodality treatment can have a profound effect on the patient management. However, these patients belong to a poor prognosis group with an overall unfavorable outcome, even with today´s standard treatment.
